# Psychometrics of the Patient Health Questionnaire (PHQ-9) in Uganda: A Systematic Review

**DOI:** 10.3389/fpsyt.2022.781095

**Published:** 2022-03-07

**Authors:** Mark Mohan Kaggwa, Sarah Maria Najjuka, Scholastic Ashaba, Mohammed A. Mamun

**Affiliations:** ^1^Department of Psychiatry, Faculty of Medicine, Mbarara University of Science and Technology, Mbarara, Uganda; ^2^African Centre for Suicide Prevention and Research, Mbarara, Uganda; ^3^Department of Internal Medicine, College of Health Sciences, Makerere University, Kampala, Uganda; ^4^CHINTA Research Bangladesh, Dhaka, Bangladesh; ^5^Department of Public Health and Informatics, Jahangirnagar University, Dhaka, Bangladesh; ^6^Department of Public Health, Daffodil International University, Dhaka, Bangladesh

**Keywords:** Patient Health Questionnaire, depression, Uganda, PHQ-9, psychometrics, systematic review, HIV, review

## Abstract

**Background:**

Depression is screened by many psychological tools, whereas the Patient Health Questionnaire-9 (PHQ-9) is one of the most commonly used self-administered tools. Uganda is a culturally diverse country with a wide variety of tribes, ethnic groups, languages, and disease conditions; it is urgent to know the psychometrics of the used PHQ-9 across different cohorts. However, there is no prior review to assess its reliability in this culturally diverse country, where this review fulfills the knowledge gap.

**Methods:**

Adhering to the PRISMA guideline, a systematic search was performed in several databases (i.e., PubMed, Africa-Wide Information, AJOL, and PsycINFO, among others), and a total of 51 articles were included in this review, confirming the study inclusion criteria (e.g., using the PHQ-9).

**Results:**

The PHQ-9 has been used among individuals above 10 years and both genders, and the tool has been used most among the HIV patient group (*n* = 28). The tool is frequently administered by interviews and has been translated into several languages (mostly Luganda, *n* = 31). A cutoff of 10 was commonly used to identify clinical or major depression (*n* = 23), and its prevalence ranged from 8 to 67%. It has been validated for use in two populations, (i) HIV-positive participants and (ii) the general population attending a health facility. The sensitivity and specificity were 92 and 89%, respectively, at a cutoff score of 10, whereas 67 and 78%, respectively, at a cutoff score of 5. The Cronbach alpha ranged between 0.68 and 0.94.

**Conclusion:**

The PHQ-9 has been used in several studies in Uganda but validated in only two populations and is commonly used in one language. Thus, validation of the tool in various populations and languages is warranted to improve the tool's acceptance in Uganda.

## Introduction

Over 300 million people worldwide suffer from depression, the single most significant factor contributing to global disability (1). In addition, it is reported that ~9 out of every ten suicide occurrences are due to mental disorders, whereas depression accounts for almost two-thirds of these cases (2). Given the adverse effects of depression, it is regularly screened by mental health workers, general practitioners, medical and surgical subspecialties, clinical officers, and nurses (3). Various methods are used to screen for depression, such as Diagnostic and Statistical Manual of Mental Disorders criteria and psychological tools. The psychological tools include Patient Health Questionnaire (PHQ), Beck's Depression Inventory (BDI), Hamilton Rating Scale for Depression, Zung Self-Rating Depression Scale (ZSRDS), Montgomery-Asberg Depression Rating Scale, Symptom Checklist-20, Center for Epidemiologic Studies-Depression Scale, Akena Visual Depression Inventory, and Mini-International Neuropsychiatric Interview (MINI) (4–7). Most of the tools are clinician-administered, but PHQ, BDI, and ZSRDS are self-administered (4).

The PHQ is the commonly used tool in Uganda, and it has various versions depending on the number of items used for depression assessment, such as PHQ-9 (9 items), PHQ-8 (8 items), PHQ-4 (4 items), and PHQ-2 (2 items) (8). Items are designed to capture the depression symptoms adhering to the Diagnostic and Statistical Manual of Mental Disorders criteria, where each item is scored from 0 to 3 (ranging from “not at all” to “nearly every day”) for responses on the experience of these symptoms within the past 2 weeks. The nine-item tool (PHQ-9) has a total score of 0 to 27, with 1–4 for minimal depression, 5–9 for minor depression, 10–14 for moderate depression, 15–19 for moderately severe depression, and 20–27 for severe depression (4, 9, 10). Based on a recent systematic review of the PHQ-9, its sensitivity and specificity for major depressive disorder (MDD) range between 37 and 98% and between 42 and 99%, respectively (11). The internal reliability of the PHQ-9 is good, with a Cronbach alpha ranging from 0.67 to 0.89 (11). Just like other psychometric tools, the accuracy of PHQ-9 depends on various factors such as (i) the administrator—self-report produces many false negatives or positives depending on participants motives, (ii) the accuracy of the translation to another language—some statements cannot be directly translated in some languages, (iii) culture acceptability of the symptoms tested—some depressive symptoms are culturally acceptable (e.g., loss of appetite among adolescent girls, or admitting to feeling sad), (iv) physiological or pathological state of the patient at the time of its administration—patients with pain and other chronic diseases commonly report sadness, insomnia, and anhedonia (12, 13).

The tool has been used in many cultures and languages, and it has persistently had good reliability. Uganda is a culturally diverse country with over 54 tribes and five ethnic groups (14). In addition, the country also has low levels of education to enable the majority of the population to comprehend the tool in its raw form—English (15). The country also has multiple refugee groups from different neighboring countries (e.g., Sudan, Democratic Republic of Congo, Rwanda, Burundi, Somali, and Ethiopia), all with different dialects (16–19), and various disease conditions whose symptoms may lead to false positives with the tool such as TB and cancers, with masked symptoms such as loss of energy, anhedonia, loss of appetite, and poor sleep (12). In line with the issues mentioned above, the accuracy of depression detection by the PHQ-9 has limited generalizability.

Although the PHQ-9 has been a widely used tool in Uganda, a culturally diverse country, no systematic evaluation assessing its reliability and psychometric properties had been performed to the best of the authors' knowledge. However, these properties typically help identify and define suitability or reliability for the use of the tool that reveals information about relevance, adequacy, and usefulness. Therefore, a systematic review was undertaken to address this gap, considering all studies that used the PHQ-9 in Uganda, which is anticipated to improve the cultural acceptance of the tool in the country.

## Methods

### Search Strategy

The guideline as provided in the Preferred Reporting Items for Systematic Reviews and Meta-Analyses (PRISMA) (20) was adhered to the present study to conduct a systematic review. A systematic literature search was performed with assistance from the university librarian (Mr. Wilson Adriko) in the databases including PubMed (*n* = 71), AJOL (*n* = 1), Cochrane library (*n* = 4), and Scopus (*n* = 48), from inception to May 10, 2021 (Supplementary Material 1). Additional searches were carried out on the databases including Google Scholar, Africa-Wide Information, PsycINFO, Global Health, Web of Science, CINAHL, and even ResearchGate for any missing articles in the primary search. The utilizing search strategy included keywords: (depression OR depress^*^ OR unipolar^*^ OR major OR “mood disorder”); AND (PHQ^*^ OR “Patient Health Questionnaire” OR PHQ-9); AND (Uganda OR Kampala). (Boolean search operators ^*^ = Words match if they begin with the word preceding the ^*^ operator; and “…” = characters return only results that contain the phrase with double quote).

### Study Selection Criteria

First of all, “Title and Abstract” of all of the retrieved literature were screened independently by MMK and SMN. Then, the full-text article was evaluated to confirm if the article adhered to the inclusion criteria. Any disagreement among the individuals was settled by the content expert (MAM). Finally, the articles were included in this review based on the inclusion criteria: being Ugandan studies using the PHQ-9, published in English as a peer-reviewed journal article, or a thesis, or a preprint article.

### Data Eligibility

**Figure 1:**
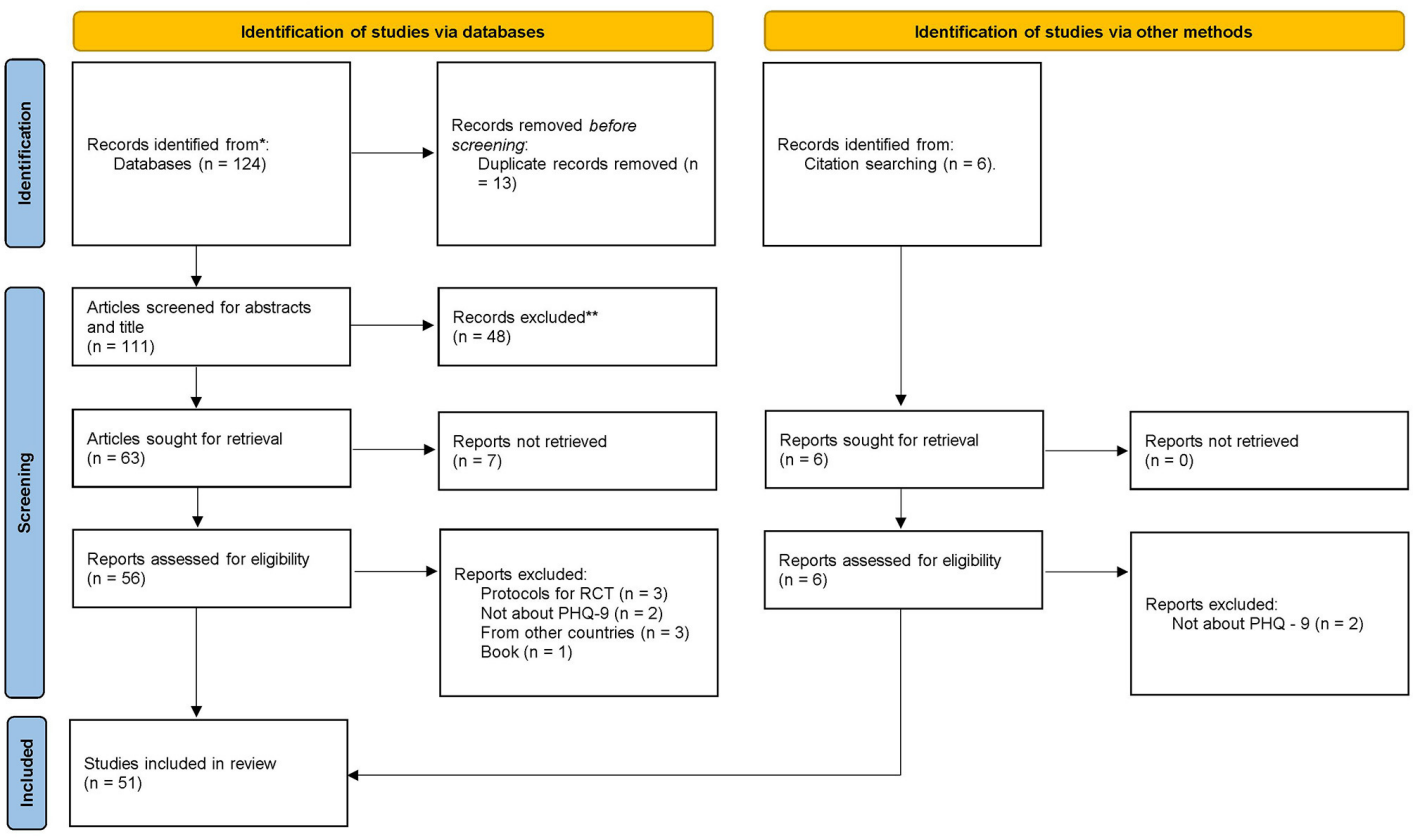
PRISMA flowchart.

A total of 124 articles were retrieved from several databases. Of these, 111 articles remained after removing duplicates and screening their “Titles and Abstracts”; a total of 48 articles were eliminated. In addition, the full text of seven articles was not possibly retrieved; although the corresponding authors were contacted, no responses were received after 1 week. However, full texts of the remaining 56 articles and six articles retrieved by citation searching were assessed for eligibility. Of these, 11 full-text articles were excluded because of the following reasons: (i) protocol for Randomized Control Trial (RCT) (*n* = 3), (ii) did not use the PHQ-9 (*n* = 4), (iii) belonged to other countries (*n* = 3), and (iv) was a book (*n* = 1). In the end, a total of 51 articles met the inclusion criteria of this review and were selected (Figure 1).

### Bias Evaluation and Quality Assessment

The Joanna Briggs Institute (JBI) checklist was used to evaluate the risk of bias of the included articles (21). The JBI uses a 4-point Likert point with the answers “no,” “yes,” “unclear,” and “not applicable,” for the following questions: (1) appropriateness of the sample frame; (2) recruitment procedure; (3) adequacy of the sample size; (4) description of subjects and setting; (5) description of the identified sample; (6) validity of the methods used to screen for depression; (7) reliability of the methods used to screen for depression; (8) adequacy of statistical analyses; and (9) response rate. Articles were assigned one point per yes, representing the score range 0–9. Articles with a score of <5 were considered low quality and were to be excluded. No article was excluded after quality assessment (Table 1).

**Table 1:** Characteristics of the included literatures in the present review.

**References**	**Rating for quality**	**Year of data collection**	**District**	**Type of study**	**Study group**	**Sample size**	**Female gender *n* (%)**	**Age**	**Education level (%)**	**Translated Language**	**Who administered the tool**	**Score cutoffs for depression**	**Prevalence of depression**	**Diagnostic standard tool**	**Psychometric measurements**
Wagner et al. (22)	9	–	Jinja, Kampala	Cohort	HIV patients	602	410 (68)	μ = 36 Range: 20–62	*P* = 54; S = 37	Luganda	Interviewer	≥10; continuous	13%; μ: 5 ± 4	–	–
Akena et al. (23)	9	2011	Kampala	Cross-sectional	HIV patients	368	261 (71)	Group: 18–71. μ: 38 ± 10	*P* = 50; S = 50	Luganda	Health worker	≥10 (screening)	–	MINI	–
Nakasujja et al. (24)	9	2008–2009	Kampala	Cohort	Patients with psychosis (HIV positive and negative)	478	276 (58)	HIV+ μ = 33 ± 8. HIV– μ = 29 ± 8	Over 7 years in educated = 372 (78)	Luganda	Interviewer	Continuous (severity of symptoms)	–	MINI	–
Tumwine et al. (25)	9	2008–2009	Jinja, Kampala	Cohort	HIV patients	602	410(68)	μ: 36 ± 9	< S:54; ≥S = 46	Luganda	Interviewer	Continuous	–	–	–
Wagner et al. (26)	9		Jinja, Kampala	Cross-sectional	HIV patients	602	355 (59)	μ: 36 ± 8. Range: (22–63)	≥S = 39	Luganda	Interviewer	Continuous	–	–	–
Wagner et al. (27)	9	2008–2009	Jinja, Kampala	Cohort	HIV patients	602	410 (68)	μ: 36 ± 9	*S* = 41	Luganda	Interviewer	Continuous, Minimal = 1–4, Mild = 5–9, Clinical = ≥10	μ: 5 ± 4. Minimal = 48%. Mild = 39%. Clinical = 13%	HSCL	–
Wagner et al. (28)	9	2008	Jinja, Kampala	Cohort	HIV patients	602	410 (68)	μ: 36 ± 9	*S* = 41	Luganda	Interviewer	Continuous	μ: 5	–	–
Walusaga et al. (29)	9	–	Kampala	Cohort	HIV patients	280	151 (54)	μ: 33	≥S = 42	Luganda	Interviewer	Continuous	μ: 4	–	–
Akena et al. (30)	8	2011	Kampala	Cross-sectional	HIV patients	368	261 (71)	μ: 31 ± 10.	*S* = 54	–	Health worker	≥10		MINI	Sensitivity = 92; sensitivity = 89; correct classification = 89 LR+ = 8.35; NR-= 0.09
Linnermayr et al. (31)	9		Jinja, Kampala	Cohort	HIV patients	602	410 (68)	μ: 36	*S* = 41	–	Interviewer	Continuous	μ:5	–	–
Okeke and Wagner (32)	9	2008–2010	Kampala	Cohort	HIV patients	482	309 (68)	μ: 35	91 had some schooling	–	Interviewer	≥10	8%	–	–
Musisi et al. (33)	9	2010–2011	Mityana, Mukono, Wakiso	Cohort	HIV patients	386	225 (58)	μ: 36 ± 9	*S* = 17	Luganda	Interviewer	Mild = 5–9; Major = ≥9	Minor: 19%; Major: 11%	–	–
Wagner et al. (34)	9	2010–2011	Kampala, Mityana, Mukono, Wakiso	Cohort	HIV patients	798	530 (66)	μ: 36 ± 9	*S* = 15	Luganda	Interviewer	Continuous	μ: 4	MINI	–
Wagner et al. (35)	9	2010–2011	Kampala, Mityana, Mukono, Wakiso	Cohort	HIV patients	798	530 (66)	μ: 36 ± 9	*S* = 15	Luganda	Interviewer	Continuous	μ: 4	–	–
Wagner et al. (36)	9	2010–2011	Jinja, Kampala	Cohort	HIV patients	1,731	1,131 (65)	μ: 36	*S* = 11	Luganda	Interviewer	Continuous; Mild = 5–9; Major = ≥9	μ: 4 ± 4; Major: 9%; Minor: 28%.	–	–
Wagner et al. (35)	9	2008–2011	Kampala, Mityana, Mukono	Cohort	HIV patients	750	475 (63)	μ: 34	*S* = 11	Luganda	Interviewer	Continuous; Mild = 5–9; Major = ≥9	μ: 4–5%; Minor = 16–37%; Major = 3–9%	–	–
Gyagenda et al. (37)	9	2014	Kampala	Cross-sectional study	Stroke patients	73	43 (58.9)	Group: 20–99	*P* = 37	Luganda	Interviewer	≥5	31%	–	–
Mwesiga et al. (38)	9	2013	Wakiso	Cross-sectional study	HIV patients	345	245 (71)	Group: 20–50+	*P* = 42	Luganda	Interviewer	≥10	17%	MINI	–
Ngo et al. (39)	9	2009–2011	Mityana, Mukono, Wakiso	Cohort	HIV patients	1,903	1,492 (78)	μ: 36 ± 9	*P* = 73	Luganda	Interviewer	Continuous. Mild = 5–9; Moderate = 10–14; Moderately severe = 15–19; Severe = >19	μ: 15 ± 5. Mild = 16%; Moderate = 36%; Moderately severe = 27%; Severe = 21%	MINI	All positively screened participants were positive on MINI
Okello et al. (40)	9	2010–2011	Kampala	Cohort	HIV patients	798	530 (66)	μ:36 ± 9	≥S = 15	Luganda	Interviewer	Continuous. ≥10	μ: 4 ± 5. 13%	–	–
Akena et al. (3)	9	2013	Luweero, Mityana, Mpigi, Wakiso	Cohort	HIV patients	1,252	961 (77)	μ: 40 ± 11. Range 18–85	*S* = 17	–	trained health workers and trained lay workers	≥10	67%	–	– Huang et al. (41)	9	2013	Kampala	Cross-sectional	parents of children in primary 3	303	248 (82)	μ: 36 ± 10. Range 18–79	*P* = 48	Luganda	Interviewer	≥10	28%	–	–
Nakku et al. (42)	9	2014	Kamuli	Cross-sectional	Patients attending a health facility	1,407	1,017 (72)	μ: 33 ± 13. Range 18–82)	*P* = 48	Luganda	Interviewer	≥5 (recommended)	≥8 = 10%	MINI	Sensitivity = 67; specificity = 78; Positive predictive value = 52; Cronbach alpha = 0.68
Wagner et al. (43)	9	2013–2014	Luweero, Mityana, Mpigi, Wakiso	RCT	HIV patients	1,252	962 (77)	μ: 40 ± 11.2	*S* = 19	Luganda	Interviewer	Continuous. Minimal = <5; Mild = 5–9; Moderate = 10–14; Moderately severe = 15–19; severe = 20–27; and Clinical = >9	μ: 8 ± 4. Minimal = 18%; Mild = 53%; Moderate = 20%; Moderately severe = 8%; Severe = 2%. Clinical = 30%	–	–
Wagner et al. (44)	9	2013–2014	Luweero, Mityana, Mpigi, Wakiso	RCT	HIV patients	1,252	962 (77)	μ: 40 ± 11.2	*S* = 19	Luganda	Interviewer	Continuous; 0–9; 10–14; and 15–27	μ: 8 ± 4; 0–9 = 70%; 10–14 = 20%; 15–27 = 10%	–	–
Wagner et al. (45)	9	2008–2011	Kampala, Mityana, Mukono, Wakiso	Cohort	HIV patients	1,021	653 (64)	μ: 36.0	*S* = 16	Luganda	Interviewer	Minor = 5–9; Major = ≥10	Minor 28%; major = 9%	–	–
Jones et al. (46)	9	2013	Kampala	Cohort	Post-tuberculosis lung disease patients	29	14 (52)	Group: 17–69. μ: 45 ± 13	*P* = 31, *S* = 31	–	Interviewer	≥5	24%	–	–
Wagner et al. (47)	9	2013	Luweero, Mityana, Mpigi, Wakiso	Cohort	HIV patients	1,252	1,028 (78)	μ: 40 ± 11	*S* = 18	Luganda	Interviewer	Continuous; Minimal = 0–4; Minor = 5–9; and Major = ≥ 10	μ: 8; Minimal = 18; Minor = 53; and Major = 30	–	–
Wagner et al. (48)	9	2011	Wakiso	RCT	HIV patients	105	86 (82)	μ: 37 ± 9	*S* = 56	Luganda	Interviewer	Continuous; Minor = 5–9; Moderate = 10–14; Moderately severe = 15–19; and severe = ≥20	μ: 17; Minor = 5%; Moderate = 27%; Moderately severe = 33%; and Severe = 31%.	MINI	–
Alinaitwe. (49)	9	–	Kampala	Cross-sectional	TB patients	308	120 (39)	Group: <30–>50	*P* = 44	Luganda	Health worker	≥10	34%	–	–
Baron et al. (50)	9	2015	Kamuli	Cohort	General population	64	–	–	–	Lusoga	Health worker	≥10	–	–	–
Beyeza-Kashesya et al. (51)	9	2013	Jinja, Kampala	Cohort	HIV clients in committed relationships and with intentions to conceive	400	299 (75)	μ: 34 ± 8	*S* = 47	Luganda	Interviewer	–	–	–	–
Kiprotich (52)^a^	9	2017	Kampala	Cross-sectional	Caregivers of children with mental illness	141	85 (60)	Group 18–50	*S* = 47	–	–	–	–	–	–
Nalwadda et al. (53)	9	2013	Kamuli	Cross-sectional	Community and facility survey men	1,129	1,129 (0)	Group: <30–≥60	*P* = 48 (C), 45 (F)	Luganda	Interviewer	Mild = 5–9; Moderate = 10–14; and Severe = 15–27	Mild = 24% (C) and 19% (F); Moderate = 3% (C) and 5% (F); Severe = 1% (C) and 0 (F)	–	–
Tol et al. (16)	9	–	Yumbe	Cohort	Refugees	55	33 (60)	μ: 35 ± 10	μ years 8 (male) vs. 3 (female)	Juba Arabic	Interviewer	–	–	–	Cronbach alpha = 0.75
Abaho et al. (54)	9	–	Kampala	Cross-sectional	Adolescents aged between 11 and 22 years	90	50 (56)	Group: 11–22	*S* = 100	–	Self-administered	–	–	–	–
Ortblad et al. (55)	9	2016	Kampala	RCT	Female sex workers	960	960 (100)	Median (IQR) =28 (24–32)	*P* = 45	–	Interviewer	>10	–	–	–
Ssebunnya et al. (56)	9	–	Kamuli	Cross-sectional	general population	1,290	848 (66)	–	–	–	Interviewer	≥10	6%	–	–
Akena et al. (57)	9		Kampala	Cohort	Diabetes	10	6 (60)	–	–	–	Health worker	≥10	–	–	–
Bahati et al. (58)^b^	9	2019	Mbale, Mbarara	Cross-sectional	Refugees	343	145 (42)	Group 14–60+	*S* = 49	–	Interviewer	≥5	96%	–	–
Kabunga (18)	5	2019	Isingiro	Cross-sectional	Refugees	146	74 (53)	Group: 18–60+	–	Kiswahili, Kinyarwanda	Interviewer	≥5	81%	–	–
Kuteesa et al. (59)	9	2017	Mukono	Cross-sectional	15–24 years individuals in a fishing community	1,281	606 (47)	Group: 15–24	*P* = 47	Luganda	Interviewer	Minimal = <5; Mild = 5–9; and Moderate to severe >9	Minimal = 88 %; Mild = 10%; and Moderate to severe = 2	–	–
Logie et al. (60)	9	2018	Kampala	Cross-sectional	Refugees 16–24 years	445	333 (75)	Group: 16–24	–	Kiswahili, French	Interviewer	Mild to moderate = 5–14; Moderately severe to severe = ≥ 15	Mild to moderate = 57%; Moderate to severe = 11%	–	–
Mahmud and Riley (61)	9	2020	Kyenjojo	Cohort	General population	1,075		–	–	–	Interviewer	≥10	14%	–	–
Olum et al. (29)	9	2019	Kampala	Cross-sectional	Medical students	331	133 (40)	Group: 18– >36+	*T* = 100	–	Self-administered	≥10	22%	–	–
Vancampfort et al. (62)	9	–	Buikwe	Cross-sectional	Fishing community	256	177 (70)	μ: 40 ± 10	75 educated	Luganda	Interviewer	Continuous	μ = 5	–	–
Wagner et al. (63)	9	2013	Kampala	RCT	HIV patients	153	92 (64)	μ: 39 ± 10	*S* = 11	Luganda	Self-administered	Continuous; Mild = 5–9; Major = ≥9	μ: 4 Minor:31%; Major: 10%	–	–
Kabunga and Anyayo (17)	4	2020	Isingiro	Cross-sectional	Refugees	146	77 (53)	Group: 18–60+	–	Kiswahili, Kinyarwanda	Interviewer	–	47%	–	–
Kabunga and Nambozo (64)	8	2020	Lira	Cross-sectional	Adolescents 10–19 years	164	87 (53)	Group: 10–19	–	Langi	Interviewer	–	34%	–	–
Kaggwa et al. (65)	6		Isingiro	Cross-sectional		153	153 (100)	μ: 33 ± 7	*P* = 48	Runyankole/ rukiga	Interviewer	≥10	65%	–	Cronbach alpha = 0.94
Ssewanyana et al. (66)	8	2018	Kampala	Cross-sectional	Patients with stomas	51	11 (21)	Group: 18–60	*P* = 47	Luganda	Interviewer	≥5	88%	–	–

a *Thesis*;

b *Preprint; P, Primary level of education; S, Secondary level of education; T, Tertiary level of education; μ, mean; SD, Standard deviation; LR+, positive likelihood ratio; LR−, Negative likelihood ratio; RCT, Randomized clinical trial; C, community sample; F, Facility sample*.

### Data Extraction

In Microsoft Excel, a data extraction file was created to extract the information from the included articles. Then, two independent reviewers extracted data on the team utilizing the following criteria: (i) first author name and publication year, (ii) year of data collection, (iii) districts where data were collected, (iv) type of study; (v) study group, (vi) sample size, (vii) gender, (viii) age, (ix) level of education, (x) translated language, (xi) administrator of PHQ-9, (xii) cutoff scores, (xiii) prevalence, (xiv) standard/confirmation tool used, and (xv) psychometric measurements. Any disagreement among the entered data was settled by the team supervisor (SA), who cross-checked and updated the final data extracted file. However, the final data extracted file is presented in Table 1.

## Results

### Description of the Included Studies

**Figure 2:**
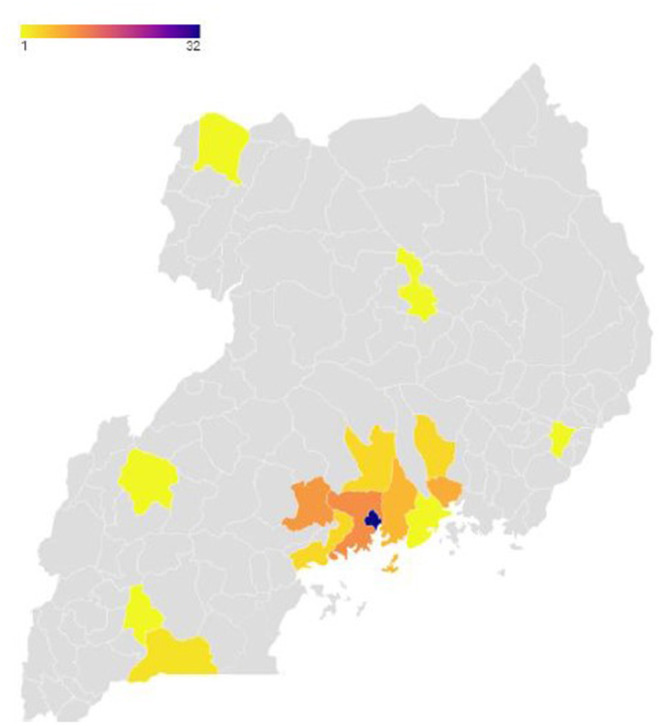
Map of Uganda showing districts where the studies were conducted.

A total of 51 articles (49 peer-reviewed, one preprint, and one thesis) were included in the review. In 24 studies, the used study design was both cohort and cross-sectional study, but only five randomized clinical trials were found. These included studies conducted between 2011 and 2021, where participants ranged from 29 to 1,903. Two studies involved participants of any of two genders [i.e., female (65) and male (53)], whereas the lowest number of female participants was 11 (66) and the highest was 1,492 (39), and the percentage of female participants ranged from 21% (66) to 82% (48). Most of the studies reported participants' mean age (*n* = 37), ranging between 31 and 45 years. One study reported median age (55), and the remaining studies (*n* = 13) used age categories. The majority of the participants of the included studies had attained education (Table 1). The studies were conducted in various parts of the country, with the majority being conducted in the capital city, Kampala (*n* = 32), and its surrounding districts. For details of the study distribution in the country (see Figure 2).

### Study Population of the Included Studies

The majority of the studies were conducted among HIV-positive participants (*n* = 28), whereas five were among refugee communities, and others were conducted among patients of other medical conditions (i.e., diabetes, TB, psychosis, and patients with a stoma or stroke). Studies were also done among the general population and community (*n* = 6). Four studies were also done among adolescents and children (age range 10–24).

### PHQ-9 Tool Administration

Trained research interviewers were used to administer the PHQ-9 tool in most studies (*n* = 42), whereas health workers administered five studies, and only three studies were self-administered (54, 63, 67). In addition, the tools were commonly administered in the translated Luganda language (*n* = 31), whereas 3 studies were in Kiswahili, 2 studies were in Kinyarwanda, and one study each was conducted for the rest of the languages, including Juba Arabic, French, Lusoga, Langi (Luo), and Runyankole/Rukiga.

### Cutoff Scores Used in the Included Studies

About 20 studies considered a continuous score for the depression symptoms and reported the corresponding mean and standard deviation, whereas most studies (*n* = 23) used a cutoff of ≥10 to indicate clinical depression or major depression. Other cutoffs included 1–4 signifying minimal depression (*n* = 4), 5–9 for minor or mild depression (*n* = 12), 10–14 for moderate depression (*n* = 5), 15–19 for moderately severe depression (*n* = 3), and 20–27 for severe depression (*n* = 3). Other unique scores included 0–9 for minimal (*n* = 1) and 5–14 for moderately severe depression (*n* = 1).

### Validation and Psychometric Properties

The PHQ-9 Cronbach alpha was reported in three studies (i.e., 0.68, 0.75, and 0.94) (16, 42, 65). The tool had mainly been validated with MINI (*n* = 2) (30, 42), but HSCL was also used (*n* = 1) (30). The tool was validated for use in two populations (368 HIV patients and 1,407 individuals of the general population), all in the same language, Luganda (30, 42). For HIV patients, the sensitivity and specificity were 92 and 89%, respectively, at a cutoff score of 10 (30), and were 67 and 78%, respectively, at a cutoff of 5 for the general population attending a health facility (42).

## Discussion

The present review summarizes the existing evidence for the use, reliability, and validity of the PHQ-9 among the studies conducted in different Ugandan cohorts. This review can be considered to provide an overview of how the PHQ-9 is being used in Uganda and guide future direction on validating the depression measuring tools in this culturally diverse country.

The PHQ-9 has been consistently found to have good psychometric properties in many parts of the globe (68). However, this is the first systematic review assessing the psychometric properties of the PHQ-9 based on the studies conducted in Uganda. The tool's sensitivity and specificity for MDD are found to be good, 92 and 89% at a cutoff score of 10, 67, and 78% at a cutoff of 5, respectively. Similar findings for this cutoff score of 10 are reported in many validation studies or systematic reviews outside the country (6, 69). The sensitivity was 68.6% [95% confidence interval (CI) 48–83.7%] and 88% (CI 77–94%) (6), while the specificity was 84.5% (CI 74.3–91.1%) (69) and 78% (CI 65–88%) (6). Despite the good psychometric properties, the results are from very few studies, making it difficult to generalize the findings in a country with diverse cultures. However, the tool has been translated to the country's different languages, with a moderate to excellent reported Cronbach alpha (0.68–0.94). Although a Cronbach's alpha of 0.70 or greater is regarded as acceptable for a self-reported instrument, the PHQ-9 (70), the studies included in this review were mainly interviewer-administered. Hence, more studies are needed to validate and make the tool culturally acceptable and understood among the different languages by adjusting a few items or developing culturally applicable tools such as a tool developed for assessing depression among adolescents living with HIV (13).

The psychometric properties of the PHQ-9 among the HIV-positive patients were found to be excellent, with a sensitivity of 92% and 100% and a specificity of 89% and 100% for the cutoff of 10 and 5, respectively (30, 39). However, the psychometric properties have been poor with the general population at a cutoff of 5 (sensitivity for MDD of 67% and specificity for MDD of 78%) (42), and the tool has not been validated in any other special group. This may be due to many studies about depression being done mainly among HIV-positive people in the country (71). Validation of the PHQ-9 in other languages, clinical or vulnerable groups, and cultures can widen the tool's application and acceptance due to its being simple to understand by patients and accuracy in detecting depression (68, 72, 73). Despite the tool not being validated in many special groups in the country, the PHQ-9 has shown good reliability at a cutoff of 10 in several groups, including (i) patients receiving psychiatry care, Cronbach alpha of 0.87, sensitivity of 93%, and specificity of 52% (74); (ii) bariatric surgery candidates; sensitivity of 75% and specificity of 76% (75); (iii) university students, internal consistency of 0.85; sensitivity of 85%, and specificity of 99% (76); (iv) patients with multiple sclerosis, Cronbach alpha of 0.82; specificity of 88%, and sensitivity of 95% (77); (v) geriatric population, internal consistency of 0.89, sensitivity of 95%, and specificity of 67% (78); and (vi) patients with psychiatric condition, Cronbach alpha of 0.88 following translation in Farsi; good correlations with PHQ-15 (0.64), and BDI-13 (0.70) (72). The tool has also been validated among several administration approaches, including *via* telephone (internal consistency of 0.91; good sensitivity of 82%, and specificity of 91%) (79) and computerized version (Cronbach alpha of 0.88, correlation with the paper version of 0.92) (80), but in review, the tool was validated for use by none of the health workers (3). The diversity of populations and special patient groups in which the tool was used, makes its reliability uncertain in multicultural countries, especially in Africa, thus requiring further research.

A number of limitations should be considered when interpreting this review's findings. First, most of the articles included in this review are from the same major study or project. The data about the reliability of the PHQ-9 also posit challenges for any possible meta-analysis. The findings are mainly from the central part of the country; thus, we cannot generalize the PHQ-9 acceptability. Despite these limitations, this review includes a large sample size, with results from both gray literature and peer-reviewed articles, showing the tools used in different populations and cultures, which are the mentionable strengths of the study. The study also included studies from different study groups that point to the generalizability of the PHQ-9 to a broader population in Uganda.

## Conclusion

The PHQ-9 has been used in several studies in Uganda but validated in only two populations and is commonly used in one language. Thus, validation of the tool in various populations and languages is warranted to improve the tool's acceptance in Uganda. In addition, with most studies using a cutoff of 10 and above for the PHQ-9, future studies are recommended to adopt this cutoff to have nationally comparable results. Because of the vast use of the PHQ-9 among participants living with HIV, the tool is suggested to be the first choice among this population due to the significant reliability and validation. However, further studies are needed among those population groups with other chronic medical conditions such as stroke, diabetes, hypertension, and TB. In addition, more studies are highly recommended to validate this tool in different languages and in different parts of the country to cater to cultural diversity.

## Data Availability Statement

The original contributions presented in the study are included in the article/Supplementary Material, further inquiries can be directed to the corresponding author/s.

## Author Contributions

MK was involved in the conception and initial manuscript drafting. MK and MM designed the study. With the assistance of a Liberian, MK identified all eligible articles from all sources and imported them to Endnote 9 to remove duplicates. SN and MK selected the remaining articles by title and then by abstract independently. MM settled any discrepancy about an included article from MK and SN. After undergoing a quality check performed by SA, MM, and MK did a final review of the articles. All authors were involved in the analysis, interpretation, substantive revisions, and approval of the final version.

## Conflict of Interest

The authors declare that the research was conducted in the absence of any commercial or financial relationships that could be construed as a potential conflict of interest.

## Publisher's Note

All claims expressed in this article are solely those of the authors and do not necessarily represent those of their affiliated organizations, or those of the publisher, the editors and the reviewers. Any product that may be evaluated in this article, or claim that may be made by its manufacturer, is not guaranteed or endorsed by the publisher.

## Abbreviations

BDI: beck's depression inventory
CI: confidence interval
MDD: major depressive disorder
MINI: mini-international neuropsychiatric interview
PRISMA: preferred reporting items for systematic reviews and meta-analyses
PHQ: patient health questionnaire
RCT: randomized control trail
ZSRDS: zung self-rating depression scale.
