# Supplementary material for: Psychometrics of the Patient Health Questionnaire (PHQ-9) in Uganda: A Systematic Review
Source: Front Psychiatry. 2022 Mar 7;13:781095. doi: 10.3389/fpsyt.2022.781095 (PMC8948461; doi:10.3389/fpsyt.2022.781095)
Supplement: Supplementary file 1 [file Data_Sheet_1.docx]

**Search strings**

PUBMED search

1. (((("depression") OR (depress*)) OR (unipolar*)) OR ("major")) OR ("mood disorder") = 1,950,156
2. ((PHQ*) OR ("Patient Health Questionnaire")) OR (phq-9) = 8,828
3. (Uganda) OR (Kampala) = 16,279
4. 1 AND 2 = 7,838
5. 4 AND 3 = 71

African Journal Online (AJOL)

1. depress* AND Uganda AND PHQ* = 1

Cochrane library

1. depress* AND Uganda AND PHQ* = 4

Scopus

1. depress* AND PHQ* AND "Uganda" AND ( LIMIT-TO ( PUBSTAGE,"final" ) ) AND ( LIMIT-TO ( AFFILCOUNTRY,"Uganda" ) ) AND ( LIMIT-TO ( EXACTKEYWORD,"Depression" ) ) AND ( LIMIT-TO ( LANGUAGE,"English" ) ) = 48
